# P-1806. ABLE to De-Label: Liberating Patients from Penicillin Chart Allergies

**DOI:** 10.1093/ofid/ofae631.1969

**Published:** 2025-01-29

**Authors:** Meaghan Martinez-Palmer, Adenike Olabode, Teena Xu, Parisa F Khan

**Affiliations:** Baylor College of Medicine, Houston, Texas; MEDVAMC, Houston, Texas; Baylor College of Medicine, Houston VA, Houston, Texas; Michael E. DeBakey Veterans Affairs Medical Center, Houston, Texas

## Abstract

**Background:**

The Veterans Affairs Clinical Pharmacy Practice Office Allergy to Beta Lactam Evaluation (ABLE) initiative was launched at the at the Michael E DeBakey VA Medical Center in October 2023. The primary implementation tool was a standardized note template, streamlining the documentation and assessment of penicillin allergy information. Patients who had tolerated penicillins since reaction or had side effects were eligible for immediate de-labeling. Those with unknown reactions, IgE-mediated allergies >10 years ago, or non-severe IgE-mediated allergies >5 years ago were considered candidates for penicillin allergy testing. All other reactions were considered ineligible for further evaluation.

Results of ABLE intervention

**Figure:**
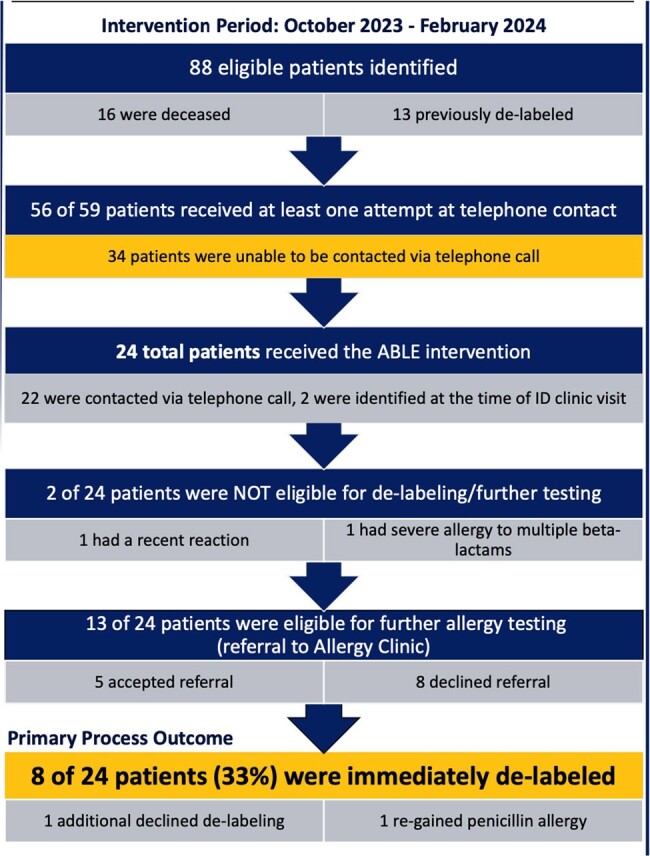

Results of ABLE intervention from October 2023-February 2024

**Methods:**

Patients labeled as penicillin allergic who were previously seen in ID clinic were contacted via unscheduled telephone calls to clarify allergy histories, identify opportunities for de-labeling, and provide recommendations for alternative beta-lactam use. The primary outcome was the proportion of “penicillin-allergic” patients successfully de-labeled.

ABLE Intervention Flowchart

**Figure:**
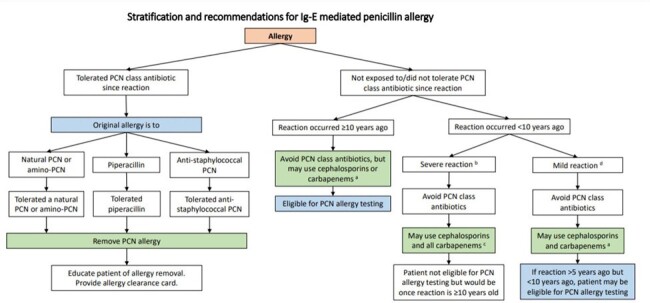

**Results:**

Of 88 patients identified, 16 (18%) were deceased and 13 (15%) were de-labeled prior to ABLE implementation in October 2023. 56 of the 59 remaining patients received at least one attempt at telephone contact. 24 patients received the ABLE intervention. Of these, 8 patients (33%) were immediately de-labeled, and 1 declined to have the allergy removed despite being eligible. Another 13 patients (54%) were eligible for further allergy testing, of which 5 (21%) accepted a referral to Allergy clinic. Two patients (8%) were not eligible for further testing. One patient who was successfully de-labeled regained the penicillin allergy label.

**Conclusion:**

The ABLE initiative improves antimicrobial stewardship and patient care by accurately assessing and managing penicillin allergies. These findings suggest those who are eligible for immediate de-labeling based on chart review should be prioritized for intervention. However, based on the acceptance rate of this telephonic intervention, alternative modalities of implementation should be explored.

**Disclosures:**

**All Authors**: No reported disclosures

